# Supplementary figures and images for: Complete Genome and Characterization Analysis of a Bifidobacterium animalis Strain Isolated from Wild Pigs (Sus scrofa ussuricus)
Source: Microorganisms. 2025 Jul 16;13(7):1666. doi: 10.3390/microorganisms13071666 (PMC12298607; doi:10.3390/microorganisms13071666)

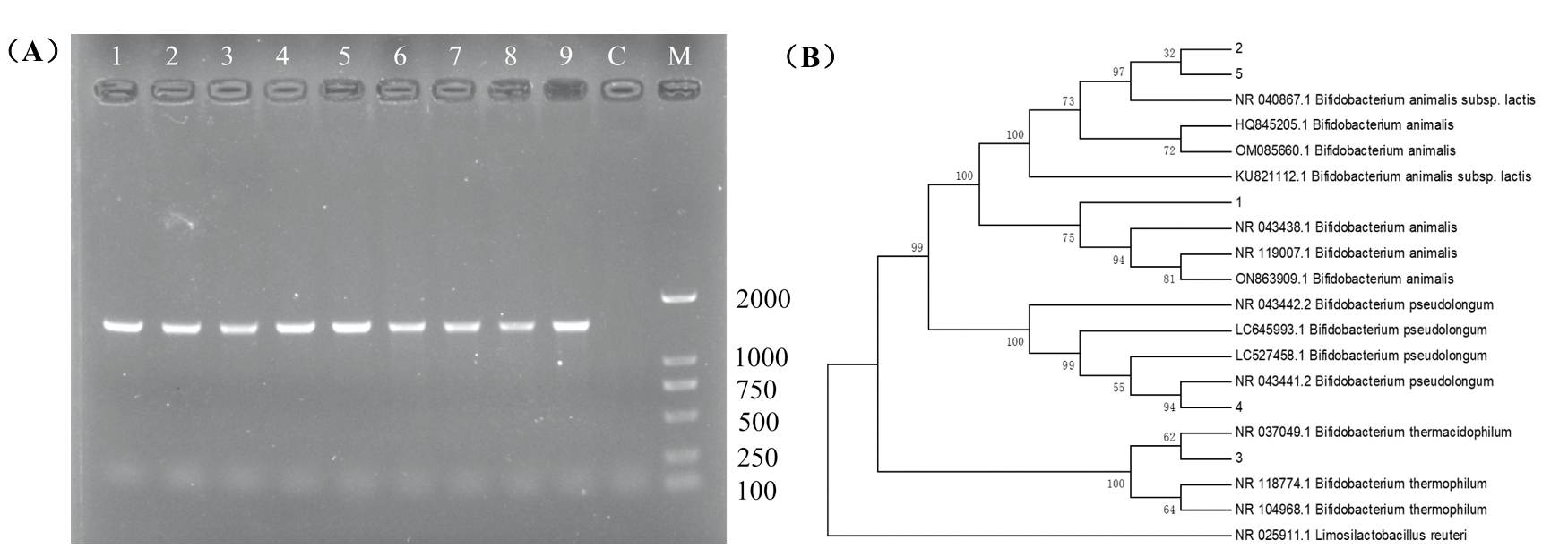

Supplement: Supplementary file 1 [file microorganisms-13-01666-s001.zip › Figure S1 Identification results of Bifidobacteria strains. .png]

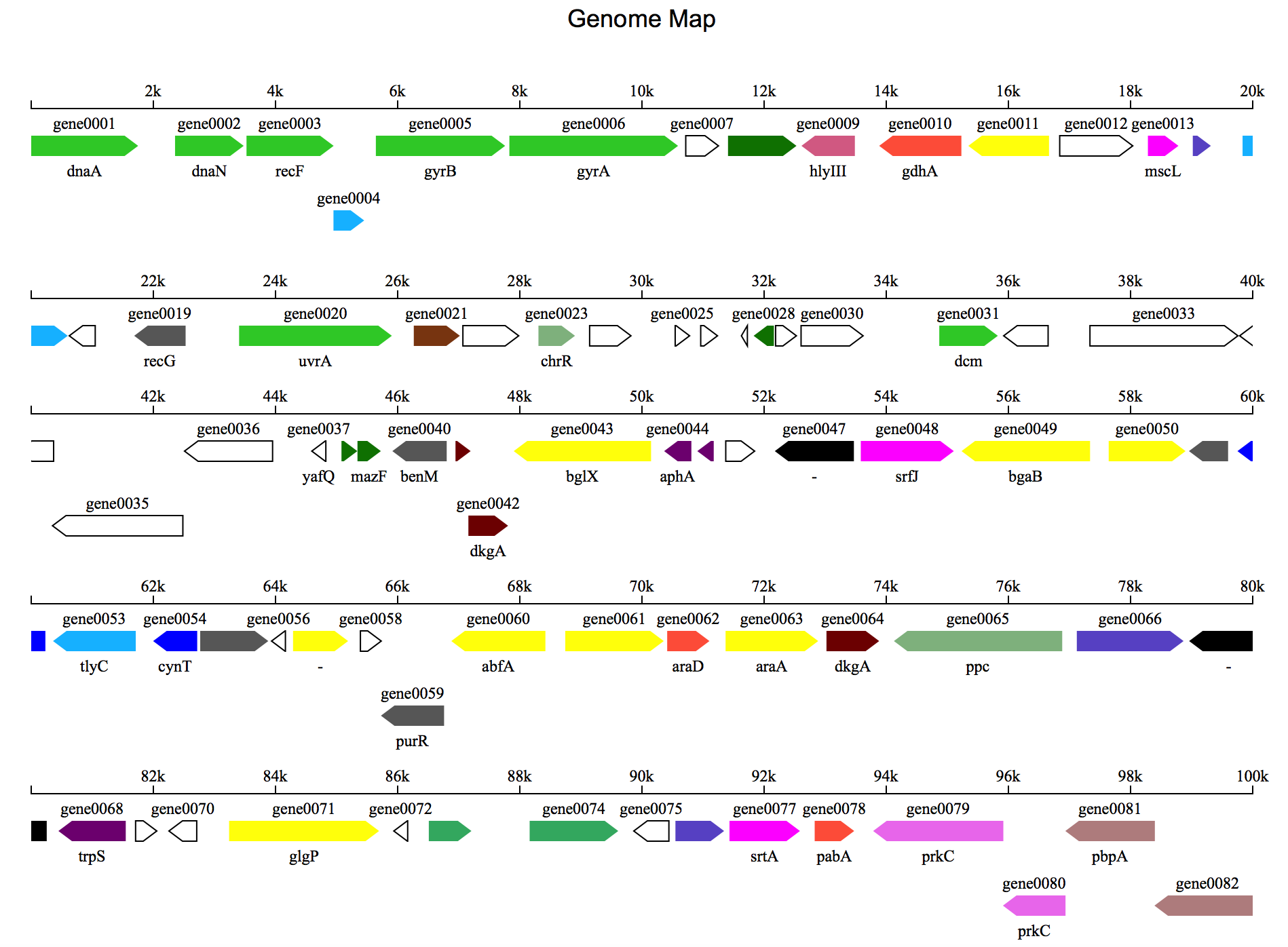

Supplement: Supplementary file 1 [file microorganisms-13-01666-s001.zip › Figure S2 Genome islands linear map.png]
